# Stress and resilience among pregnant teenagers in Ile-Ife, Nigeria

**DOI:** 10.18332/ejm/134181

**Published:** 2021-03-31

**Authors:** Aanuoluwapo O. Olajubu, Grace O. Omoloye, Temitope O. Olajubu, Adekemi E. Olowokere

**Affiliations:** 1 Department of Nursing Science, College of Health Sciences, Obafemi Awolowo University, Ile-Ife, Nigeria; 2 Department of Nursing Services, Obafemi Awolowo University Teaching Hospitals Complex, Ile-Ife, Nigeria; 3 Seventh-Day Adventist Hospital, Ile-Ife, Nigeria

**Keywords:** teenage pregnancy, adolescent, pregnancy-related stress, resilience, stigmatization, social support

## Abstract

**INTRODUCTION:**

The period of pregnancy is associated with some level of physical, emotional and psychological stress which can be particularly heightened and have more deleterious impact when the expectant mother is a teenager who needs higher level of resilience to cope with the challenges linked with motherhood. This study aimed to assess the level of perceived pregnancy-related stress and its relationship with the level of resilience.

**METHODS:**

An analytical cross-sectional study design was employed using a structured questionnaire and the study was conducted among 241 adolescents. Perceived stress and resilience were measured using Perceived Stress Scale, and Wagnild & Young Resilience Scale, respectively. Descriptive and inferential statistics were computed using percentages, means with standard deviations, Student’s t-test, Pearson correlation, one-way analysis of variance (ANOVA) and multivariate logistic regression.

**RESULTS:**

Majority of the respondents (194; 80.5%) were categorized as having moderate level of perceived pregnancy-related stress and 186 (77.2%) had low level of resilience. A significant inverse relationship was found between perceived pregnancy-related stress and resilience (r=-0.15, p=0.02). At multivariate level, three variables emerged as independent predictors of higher level of pregnancy-related stress: feeling of shame (OR=3.39; CI: 1.01–11.34), male partner’s rejection of pregnancy (OR=3.43; CI: 1.45–8.12) and lack of parental involvement in care (OR= 3.56; CI: 1.65–7.71).

**CONCLUSIONS:**

There is a significant inverse relationship between perceived pregnancyrelated stress and resilience among teenagers in Nigeria, with higher resilience among the older age groups and those who had support from significant others.

## INTRODUCTION

The period of adolescence is a very critical phase of life during which an individual undergoes several significant physical, psychological, and cognitive changes that bring both dangers and prospects that can influence the life of the adolescent^1^. Pregnancy is a period of enormous biological, psychological and social challenges. It can also be a time of emotional and psychological disturbances when dealing with its new demands; hence, stress and anxiety are common during pregnancy, especially among unmarried teenagers^2^.

In developing regions, approximately 21 million girls aged 15–19 years become pregnant yearly and about 12 million give birth. Also, an estimated 2.5 million girls aged ≤16 years give birth annually^3^. According to the UNFPA report, of the ten countries with the highest rates of adolescent pregnancy worldwide, nine are African^4^. A meta-analysis reported a higher prevalence of adolescent pregnancy in Africa compared to other low- and middle-income countries (LMIC)^5^. In Nigeria, the majority of adolescents are sexually active and involved in unprotected sexual activities^6^ with a pooled prevalence of adolescent pregnancy ranging from 1.62 to 51%^5^.

Adolescent pregnancy has been associated with several negative consequences for the teenage girls, their children, families, the country’s development and it is seen as a major public health burden, especially in Africa^5,7^. It has also been linked to increased adverse maternal and neonatal outcomes with social, educational and economic consequences^5,8-10^.

Stress is an important risk factor for the physical and psychological health of pregnant women^11^. Stress and anxiety disorders during pregnancy do not only have undesirable effects on the progression of pregnancy, they can also impact the outcome for both mother and child^2^. However, among teenagers, the effects can be particularly heightened and have more deleterious impact. It has been documented that perinatal depression is more prevalent among pregnant women and nursing mothers who are adolescents than their counterparts who are of older age^12^. Pregnant adolescents are susceptible to depression as a result of the mental stress and psychological difficulties they are exposed to during pregnancy^13^. Also, there has been indications from Nigeria that suggest poorer outcomes for children born to adolescents with depression^14^.

Common stressors among pregnant adolescents include shame and stigmatization, which result in loneliness, dropping out of school, and lack of support from loved ones^7^. Also, factors such as age, marital status, literacy levels and parity play major roles in the determination of pregnancy-related stress and anxiety levels^2^. Most pregnant teenagers live with guilt and feeling of selfcondemnation and disappointment to their parents who may eventually shoulder the responsibility of taking care of another person^15^. These pose a heavy psychological burden on pregnant teenagers with the resultant effect on their physical and mental health due to absence of capability (i.e. resilience) to cope appropriately with the unexpected life events experienced by many pregnant teenagers^3,16^.

Resilience denotes a set of personal resources that protect an individual from the negative effects of stressors, bringing about positive outcomes in the presence of adversity^17^. It has been documented as a protective factor against stress in pregnancy, puerperium and the resultant adverse maternal and neonatal outcomes in developing and developed countries^11,18,19^. However, there is paucity of data on its relationship with pregnancy-related stress among adolescents in Nigeria.

This study, therefore, assessed the level of perceived pregnancy-related stress and level of resilience among pregnant teenagers in Ile-Ife, Osun State, Nigeria. This is with the aim of providing necessary empirical evidence for advocacy towards sensitizing concerned stakeholders about the plights of pregnant teenagers in low- and middle-income countries, especially Nigeria. This will, in turn, stimulate action and promote appropriate intervention programs for better outcomes, good health and well-being.

## METHODS

### Study design

An analytical cross-sectional study design was used to determine the pattern of perceived pregnancy-related stress and resilience among pregnant teenagers in the study setting.

### Setting and participants

The study was conducted in Ile-Ife, an ancient city in Osun State, South-West Nigeria. Ile-Ife, with a population of about 0.5 million^20^, serves as the administrative and geopolitical headquarters of Osun East Senatorial district, one of the three senatorial districts in the State. There are eight Local Government Councils (LGCs) under Ile-Ife as a whole – three Local Government Areas and five Local Council Development Areas^21^. All pregnant teenagers (aged 10–19 years) attending the primary healthcare centers (PHCs) across the various Local Government Councils in Ile-Ife were the target population. The study was carried out in 20 of the 46 PHCs located in the selected Local Government Councils.

Cochran’s formula (n=Z^2^pq/d^2^) for single proportion was used to calculate the sample size^22^. Using 10% attrition rate and a previous 23% prevalence of teenage pregnancy^23^, the calculated sample size was 302. A multistage sampling technique was used to recruit respondents as follows: four LGCs were randomly selected out of the eight LGCs in Ile-Ife. In each of the four selected councils, five PHCs with the highest number of pregnant teenagers registered for antenatal care were purposively selected, yielding a total of twenty PHCs which were included in the study. The total number of respondents recruited from each of the 20 selected PHCs was proportionately determined. Ethical approval (IPHOAU/12/1192) was obtained. Informed consent or assent was secured from all participants depending on their age, along with consent from the parents/guardians of participants aged ≤18 years. The privacy and confidentiality of participants’ identity and data were ensured.

### Data collection procedure

Data were obtained with a pre-tested structured questionnaire which had four sections. The first section elicited information on sociodemographic characteristics. The second section assessed the perceived pregnancyrelated stress using a standardized and validated Perceived Stress Scale^24^. It consisted of 10 items, graded on a 5-point Likert scale (0=never to 4=very often), thus yielding a total obtainable score ranging 0–40, higher scores indicating a higher level of perceived stress. Four of the items (items 4, 5, 7 and 8) were reverse scored. The computed scores were categorized into three levels of perceived stress: low (1–13), moderate (14–26) and high (27–40)^25^. The third section assessed respondents’ emotional experiences in which they were asked to rate how often they had each of the five specific emotional experiences, i.e. feelings of shame, guilt, loneliness, helplessness and sense of stigmatization. Each emotional feeling was rated: 0=never to 3=always. The fourth section measured the level of resilience among the pregnant teenagers using the standardized 14-item Wagnild and Young Resilience Scale rated on a 7-point Likert scale (1=strongly disagree to 7=strongly agree) with higher scores reflecting higher resilience^26^. Based on standard classification, the summed scores were categorized into levels of resilience as follows: low (≤64), moderate (65–81) and high (≥82)^26^. The face validity was ensured by giving the instruments to experts in maternal and child health nursing and in the field of psychology to assess the suitability of the content of the test items and how clearly the items on the instrument reflect the concept that they are intended to measure.

The instrument was pre-tested among 15 randomly selected pregnant teenagers at the antenatal clinics in Obafemi Awolowo University Teaching Hospitals Complex, Ile-Ife. The clarity and comprehension of the items by respondents were ascertained. Reliability test for each of the scales yielded the following Cronbach’s alpha coefficients: perceived stress scale (0.70), resilience scale (0.80) and the emotional experience scale (0.92).

### Statistical analysis

Data analysis was performed using SPSS version 20. The scoring and categorization of responses in the various sections were done as described earlier. The results were summarized with frequencies and percentages for categorical variables, and means with standard deviations for quantitative variables.

Using inferential statistics, the association between continuous and categorical variables was assessed with the use of independent Student’s t-test and one-way analysis of variance (ANOVA). The relationship among continuous variables was determined using Pearson’s correlation coefficient.

The variables that were significantly associated with perceived pregnancy-related stress were entered into a multivariate logistic regression with the aim of determining the independent predictors of high perceived pregnancyrelated stress levels among the respondents. For the purpose of the regression analysis, some variables were dichotomized by combining their categories in view of the low frequencies of some categories. The perceived pregnancy-related stress variable was dichotomized into ‘low/moderate’ and ‘high’. For all analyses, the level of statistical significance was set at p<0.05.

## RESULTS

A total of 302 teenagers were approached out of which 241 respondents gave assent/consent to participate yielding a response rate of 80%.

### Sociodemographic characteristics

The age of the respondents ranged 14–19 years with a mean of 17.5±1.4 years. About two-thirds (162; 67.2%) were single while the remaining 32.8% were reportedly married.

With regard to educational status, an overall majority of 217 (90%) had formal western education, of which about half (115; 53.0%) had secondary school education while (85; 39.2%) and (17; 7.8%) had primary and tertiary education, respectively. Also, about two-third (155; 64.3%) of the respondents had no source of income (Table 1).

**Table 1:** Sociodemographic characteristics the participants

*Characteristics*	*n*	*%*
**Age** (years)		
14–16	43	17.8
17–19	198	82.2
**Marital status**		
Single	162	67.2
Married	79	32.8
**Education type**		
Informal	24	10.0
Formal	217	90.0
**Personal monthly income**, (NGN)		
No income	155	64.3
<18000	64	26.6
≥18000	22	9.1
**Living place**		
Alone/with friends	16	6.6
With parents	87	36.1
With extended family	26	10.8
With male partner	91	37.8
With partner’s family	21	8.7
**Partner accepted pregnancy**		
No	78	32.4
Yes	163	67.6
**Parents involved in care**		
No	66	27.6
Yes	175	72.6
**Peer influence**		
No	116	48.1
Yes	125	51.9

NGN: 10000 Nigerian Naira about 24 US$.

### Pattern of perceived pregnancy-related stress and resilience

The perceived pregnancy-related stress score among the respondents ranged 9–34 with a mean of 21.6±4.4. The majority (194; 80.5%) were categorized as having moderate level of perceived stress, 38 (15.8%) had a high level, while only 9 (3.7%) had a low level of perceived stress.

With regard to the degree of resilience, the total resilience scores obtained by the respondents ranged 26–87 with a mean score of 56.5±11.3. Among them, 186 (77.2%) had low resilience, 50 (20.7%) had a moderate level, while just 5 (2.1%) had a high level of resilience.

### Pattern of negative emotional experiences

The extent to which the respondents experienced some specific emotions are shown in Table 2. A total of 65% sometimes or always experienced the feeling of shame, thus it was the most prevalent of the emotions assessed. This was followed by loneliness (60.6%), stigmatization (60%), guilt (58.9%) and feeling of helplessness (51.5% ).

**Table 2:** Pattern of specific emotional experience among respondents

*Emotional experience*	*Always n (%)*	*Sometimes n (%)*	*Rarely n (%)*	*Never n (%)*
Shame	89 (36.9)	68 (28.2)	29 (12.0)	55 (22.8)
Guilt	66 (27.4)	76 (19.5)	47 (19.5)	52 (21.6)
Loneliness	68 (28.2)	78 (32.4)	50 (20.7)	45 (18.7)
Helplessness	37 (15.4)	87 (36.1)	61 (25.3)	56 (23.2)
Stigmatization	81 (33.6)	66 (27.4)	50 (20.7)	44 (18.3)

### Relationship between perceived pregnancy-related stress, resilience and emotional experiences

The correlation analysis between perceived pregnancyrelated stress and resilience scores was determined. The Pearson’s correlation coefficient showed a significant inverse relationship between perceived pregnancy-related stress and resilience, as those with higher resilience reported a low level of stress (r=-0.15, p=0.02).

With increasing age, there was a decrease in the perception of pregnancy-related stress (r=-0.15, p=0.02), while age was positively correlated with resilience (r=0.31, p<0.001). Furthermore, there was positive correlation between pregnancy-related stress and each of the emotional experience rating scores, i.e. shame (r=0.51, p<0.001), guilt (r=0.51, p<0.001), loneliness (r=0.54, p<0.001), helplessness (r=0.45, p<0.001) and feeling stigmatized (r=0.47, p<0.001).

### Relationship between perceived pregnancy-related stress and biosocial variables

In the context of demographic characteristics, the mean of perceived pregnancy-related stress scores was higher among the following category of respondents: those in the younger age group of 14–16 years (p=0.03), those who were not married (p<0.001), those who had formal education (p=0.01) and those who were not earning any income (p=0.01) (Table 3).

**Table 3:** Relationship between perceived stress and biosocial variables

*Variables*	*n (%)*	*Perceived stress scores mean (SD)*	*Test statistic t-test/ANOVA*	*p*
**Age** (years)				
14–16	43 (17.8)	22.90 (4.17)	2.24	0.03*
17–19	198 (82.2)	21.27 (4.40)		
**Marital status**				
Single	162 (67.2)	22.59 (4.10)	5.53	<0.001*
Married	79 (32.8)	19.44 (4.25)		
**Education type**				
Informal	24 (10.0)	18.62 (3.68)	-3.53	0.01*
Formal	217 (90.0)	21.89 (4.35)		
**Personal monthly income**, (NGN)				
No income	155 (64.3)	22.00 (4.49)	4.58	0.01*
<18000	64 (26.6)	21.35 (3.97)		
≥18000	22 (9.1)	19.05 (4.16)		
**Living place**				
Alone/with friends	16 (6.6)	21.94 (3.21)	10.79	<0.001*
With parents	87 (36.1)	23.38 (4.14)		
With extended family	26 (10.8)	21.58 (3.87)		
With male partner	91 (37.8)	19.47 (4.29)		
With partner’s family	21 (8.7)	22.76 (3.65)		
**Partner accepted pregnancy**				
No	78 (32.4)	23.58 (3.93)	5.19	<0.001*
Yes	163 (67.6)	20.60 (4.29)		
**Parents involved in care**				
No	66 (27.6)	22.79 (3.04)	2.70	0.01*
Yes	175 (72.6)	21.10 (4.73)		
**Peer influence**				
No	116 (48.1)	20.56 (4.08)	-3.48	0.01*
Yes	125 (51.9)	22.49 (4.49)		

NGN: 10000 Nigerian Naira about 24 US$.

* Statistically significant at p<0.05.

Also more than one-third of the respondents (91; 37.8%) were living with their male partners, followed by 87 (36.1%) living with their parents while 6.6% lived alone. The highest level of perceived stress was found among teenagers who lived with their parents and lowest among those who lived with their partners.

Also, close to one-third (32.4%) reported that their male partners did not accept their pregnancy and such respondents had significantly higher perceived stress scores (p<0.001). In the same vein, the 66 (27.6%) pregnant teenagers whose parents were not involved in their care exhibited higher perceived stress level (p=0.01). With regard to the role of peer influence, more than half (51.9%) admitted that it contributed to the events leading to getting pregnant and this was also significantly associated with the level of stress (p=0.01).

### Relationship between resilience and biosocial variables

As highlighted in Table 4, the level of resilience was found to be significantly higher among the older age group (p<0.001), those who were married (p=0.02), those who lived with their partners (p<0.001) and those whose pregnancies were accepted by their partners (p=0.02).

**Table 4:** Relationship between resilience and biosocial variables

*Variables*	*Resilience mean (SD)*	*Test statistic t-test/ ANOVA*	*p*
**Age** (years)			
14–16	50.53 (11.21)	-3.95	<0.001
17–19	57.79 (10.88)		
**Marital status**			
Single	54.95 (10.58)	-3.11	0.02
Married	59.67 (11.99)		
**Education type**			
Informal	55.13 (12.28)	-0.63	0.53
Formal	56.65 (11.16)		
**Personal monthly income**, (NGN)			
No income	55.82 (11.16)	1.02	0.36
<18000	58.20 (11.77)		
≥18000	56.32 (10.43)		
**Living place**			
Alone/with friends	58.50 (11.07)	6.04	<0.001
With parents	54.44 (10.94)		
With extended family	50.12 (11.46)		
With male partner	60.26 (10.94)		
With partner’s family	55.10 (8.41)		
**Partner accepted pregnancy**			
No	53.97 (13.08)	2.43	0.02
Yes	57.71 (10.10)		
**Parents involved in care**			
No	56.61 (12.19)	0.09	0.93
Yes	56.46 (10.93)		

NGN: 10000 Nigerian Naira about 24 US$.

### Predictors of perceived pregnancy-related stress

The various demographic, emotional and social factors that were significantly associated with perceived pregnancyrelated stress were entered into a multivariate logistic regression analysis with dichotomized perceived stress level as the dependent variable (Table 5). The perceived pregnancy-related stress levels were dichotomized into ‘low/moderate’ and ‘high’.

**Table 5:** Logistic regression analysis of predictors of perceived pregnancy-related stress

*Variables*	*Beta*	*p*	*OR*	*95 % CI*
**Resilience**				
Moderate/high	0.45	0.27	1.56	0.71–3.45
Low			1	
**Age** (years)				
17–19	0.32	0.48	1.38	0.57–3.35
14–16			1	
**Marital status**				
Single	0.01	0.99	1.01	0.44–2.30
Married			1	
**Education type**				
Formal	1.04	0.08	2.84	0.88–9.22
Informal			1	
**Income status**				
No income	-0.61	0.27	0.54	0.18–1.61
Some income			1	
**Feeling of shame**				
Sometimes/always	1.22	0.04*	3.39	1.01–11.34
Rarely/never			1	
**Feeling of guilt**				
Sometimes/always	0.31	0.54	1.37	0.50–3.73
Rarely/never				
**Loneliness**				
Sometimes/always	0.63	0.12	1.88	0.84–4.21
Rarely/never			1	
**Helplessness**				
Sometimes/always	-0.12	0.75	0.89	0.42–1.86
Rarely/never			1	
**Felt stigmatized**				
Sometimes/always	-0.24	0.70	0.79	0.24–2.58
Rarely/never			1	
**Living place**				
With partner	0.49	0.27	1.64	0.68–3.96
Others			1	
**Partner’s acceptance**				
No	1.23	0.01*	3.43	1.45–8.12
Yes			1	
**Parental involvement**				
No	1.27	0.01*	3.56	1.65–7.71
Yes			1	
**Peer influence**				
No	-0.32	0.36	0.72	0.37–1.44
Yes			1	

* Statistically significant at p<0.05.

There were three variables that emerged as independent predictors of higher level of perceived pregnancy-related stress among the respondents. These include: feeling of shame (OR=3.39; CI: 1.01–11.34), male partner’s rejection of pregnancy (OR=3.43; CI: 1.45–8.12) and lack of parental involvement in care (OR=3.56; CI: 1.65–7.71).

## DISCUSSION

The aim of this study was to assess perceived pregnancyrelated stress, its associated factors and its relationship with levels of resilience among pregnant teenagers.

The results show that there is a high prevalence of perceived pregnancy-related stress among the respondents. This is in consonance with the consistent submissions by various authors indicating that pregnant teenagers experience high level of emotional and psychological stress due to diverse psychosocial issues in addition to specific pregnancy-associated challenges^27,28^. Teenage mothers have an increased tendency of difficulty in coping with the psychological, social and economic demands that come with motherhood along with the demands of navigating the developmental challenges associated with adolescence^28,29^. More so, many teenage pregnancies are unplanned and out of wedlock with attendant social-cultural issues, thus, becoming a potentially stressful life event^28^.

In many parts of the world, including Nigeria, teenage pregnancy out of wedlock tends to be associated with social disapproval with consequent stigmatization, feeling of shame, guilt and other negative emotions^28,30^. These negative feelings, i.e. shame, guilt, helplessness, loneliness and sense of stigmatization, were quite prevalent among the respondents in this study. As emphasized by previous authors^29,30^, such emotional feelings are not only common, they increase the overall level of the stress experienced by teenage mothers. This is corroborated by the significant correlation between each of the emotional feelings and perceived pregnancy-related stress among the respondents in this study.

At bivariate level, perceived pregnancy-related stress was significantly lower among those who were married in comparison with those who were single and this is consistent with the finding in a previous study in Southern Brazil^28^. This might be expected since pregnancy in married adolescents is culturally accepted. Also, those married may benefit from the support possibly provided by their spouses, hence, mitigating the level of their perceived pregnancyrelated stress^28^. Furthermore, the level of resilience was higher among the married respondents in this study, thus, potentially acting as a buffer against the level of perception of stress and its effects.

In a similar vein, perceived pregnancy-related stress was significantly lower among those whose pregnancies were accepted by their partner and those whose parents were involved in their care. This further suggests the crucial importance of social support as a positive moderating factor on the level of stress perceived or experienced by pregnant teenagers^28^. This may also explain the significantly higher level of resilience reported by those whose pregnancies were accepted by their partners and those who lived with their partners. A number of previous authors have reported an inverse relationship between the availability of such social support and perceived stress^27,28,31^. On the other hand, a previous study^32^ did not find any relationship between stress during pregnancy and support within the family. However, the authors included adult pregnant women in their study and this may explain the discrepancy in their result compared to the present study.

Furthermore, in reference to their place of abode, it is noteworthy that those living with their parents reported the highest level of perceived pregnancy-related stress. This appears counter-intuitive considering the fact that parents would have been expected to be potential sources of the social support needed by these teenagers and the availability of such social support would have provided great relief. Therefore, this finding may suggest that some parents did not provide the kind of social and emotional support that would have achieved the reduction in the level of stress experienced or perceived by their pregnant teens. This is in support of a previous finding which showed that many parents displayed negative attitudes and dispositions towards their teenagers who got pregnant out of wedlock^30^. Such dispositions are actually potential sources of increase in the level of stress felt by such teenagers. However, a previous study in Nigeria reported social support for pregnant teenagers by their parents, especially mothers, despite the cultural and religious views^33^.

With regard to age, it was not surprising that the degree of perceived stress was higher among those in the younger age group. This may be attributable to the fact that, as shown in the results, the level of resilience is generally lower among the younger ones, which corroborates a previous similar finding^31^.

Assessment of the levels of resilience of the pregnant teenagers in this study showed that the majority had low resilience. This finding corroborates a previous study among pregnant adolescents where their level of resilience was found to be low compared with a pregnant adult^34^. The reason for this may not be farfetched as many of the teenagers are not yet physically, psychological, emotional prepared for pregnancy and associated experiences. The participants with higher resilience perceived themselves to be relatively less stressed. The experience of negative emotions such as feelings of stigma, shame, guilt, helplessness and loneliness was also significantly lower among those with high resilience. This finding was comparable to a previous study where resilience has been reported to help increase pregnant teenagers’ capacity to overcome adversities from a crisis situation such as the damaging effects of unintended pregnancies^35^. Resilience is also an enabling factor for healthcare service engagement among young mothers even in the face of stigma or negative stereotype from healthcare providers^14^. The converse relationship found between stress and resilience in this current study is understandable and agrees with findings from previous studies^11,36^. Resilience, which has been described as capacity for adapting successfully to challenging or threatening circumstances, is known to be a protective factor against the effect of stress and stressful life events^11^. Thus, helping to improve pregnant teenagers’ resilience may help improve their lives and promote opportunities for positive outcomes among them^34^.

The significant factors that were associated with perceived pregnancy-related stress at bivariate level were subjected to multivariate level of analysis. There were three factors that emerged as independent predictors of high stress levels among the respondents: feeling of shame, lack of parental involvement in care, and partner’s rejection of the pregnancy.

### Limitations

The restriction of the study setting to primary healthcare facilities potentially limits the ability to generalize the findings to the entire population or to teenagers in another sociocultural milieu. The use of a structured questionnaire also possibly confines the respondents to picking their answers from the provided options. Future studies on the subject may want to employ a qualitative method with openended questions. It must also be noted that the presence of other stressful life events was not evaluated. Such stressors are potential confounders that could influence the perceived pregnancy-related stress and resilience levels of the respondents. The study is also limited by its design and cause-and-effect relationships could not be predicted. However, the associations found between the variables in this study could be ascertained through a more rigorous study using an experimental design.

The findings in this study remain pertinent and highlight the crucial need to pay attention to the psychological wellbeing of pregnant teenagers. Efforts should be made by healthcare workers to incorporate assessment of mental wellness into their routine antenatal care. Appropriate intervention programs aimed at improving psychological health of pregnant teenagers should be designed and deployed. Such interventions should include training the pregnant teenagers on positive coping strategies and enhancement of their capacity for resilience. Parents, guardians and family members of these teenagers should also be educated on the need to provide them with adequate social support.

## CONCLUSIONS

This study shows an inverse relationship between levels of perceived pregnancy-related stress and resilience. The level of perceived pregnancy-related stress was higher among those in the younger age group and the majority had low resilience. There may be a need for policy makers and relevant stakeholders to put measures in place to address psychological needs among pregnant teenagers.
